# Videos on Bilibili, TikTok, and Xiaohongshu as Sources of Medical Information on Adenoid Hypertrophy: Cross-Sectional Content Analysis

**DOI:** 10.2196/82923

**Published:** 2026-06-18

**Authors:** Chunrong Huang, Yongfeng Zou, Liyi Zhong, Shuijiao Chen, Jingchun Lin, Yingxia Luo, Wenpin Jiang

**Affiliations:** 1 Department of Otorhinolaryngology-Head and Neck Surgery, Zhujiang Hospital of Southern Medical University, 253 Middle Gongye Avenue, Haizhu District, Guangzhou, China, 86 020-62783136; 2 Nursing Department, Zhujiang Hospital of Southern Medical University, Guangzhou, China

**Keywords:** adenoid hypertrophy, short videos, health information, quality assessment, reliability

## Abstract

**Background:**

The clinical diagnosis rate of adenoid hypertrophy (AH) in children has increased in recent years, drawing growing attention from parents. Short-video platforms such as Bilibili, TikTok, and Xiaohongshu host a large volume of educational content on this condition. However, the quality and reliability of this information remain unclear.

**Objective:**

This study aimed to evaluate the completeness, understandability, actionability, reliability, and overall quality of short videos on AH across Bilibili, TikTok, and Xiaohongshu and to explore factors associated with these quality metrics, including uploader characteristics and engagement indicators.

**Methods:**

We collected 220 videos (Bilibili: n=90, 40.9%; TikTok: n=63, 28.6%; and Xiaohongshu: n=67, 30.5%) using newly registered accounts. Two independent reviewers evaluated video quality using a 6-item content completeness scale (score range 0-12), the Patient Education Materials Assessment Tool for Audiovisual Materials, the modified DISCERN instrument, and the Global Quality Scale (GQS). Interrater reliability was high (Cohen κ=0.77-0.993). Completeness assessed essential informational components of AH. As data were nonnormally distributed, results are presented as median (IQR). Cross-platform comparisons were conducted using the Kruskal-Wallis *H* test with post hoc Mann-Whitney *U* tests (with Bonferroni correction). Spearman correlation was used to explore associations between video characteristics (ie, duration and engagement metrics) and quality outcomes. Stepwise linear regression identified independent predictors of overall quality (GQS).

**Results:**

Video duration differed significantly across platforms (Bilibili: median 113.5, IQR 66.5-271.5 seconds; TikTok: median 73, IQR 44-100 seconds; and Xiaohongshu: median 63, IQR 41-127.5 seconds; *P*<.001). Bilibili videos demonstrated higher completeness than videos on the other 2 platforms (Bilibili: median 2, IQR 1.5‐4.0; TikTok: median 1.5, IQR 0.5‐2.0; and Xiaohongshu: median 1.5, IQR 0.5-2.8; *P*<.001); overall differences were observed for understandability and reliability, but pairwise comparisons did not reach statistical significance after Bonferroni correction. Xiaohongshu videos showed greater actionability than TikTok videos (*P*=.011). Medical professionals (n=158, 71.8%) had higher understandability than nonprofessionals (n=158, 81.8% vs n=62, 66.7%; *P*=.001). Video duration positively correlated with completeness (ρ=0.64, 95% CI 0.56-0.71; *P*<.001). Shares showed weak positive correlations with completeness and actionability. Stepwise regression identified understandability (using the Patient Education Materials Assessment Tool–Understandability) as the strongest independent predictor of overall quality (GQS), followed by actionability, video duration, and uploader type; engagement metrics and platform did not enter the final model.

**Conclusions:**

The quality of AH-related videos on Chinese short-video platforms is generally suboptimal. Bilibili offers higher completeness, while Xiaohongshu excels in actionability and interactivity. Understandability is the strongest predictor of overall quality, surpassing uploader type and engagement metrics. To improve online health information, platforms should move beyond engagement-based algorithms, and health care professionals should prioritize clear, actionable content.

## Introduction

Adenoid hypertrophy (AH) is a common pediatric condition, affecting approximately 34.5% of children, although prevalence estimates may vary depending on the age group and diagnostic criteria [1]. AH can obstruct the Eustachian tube, leading to otitis media, hearing loss, and tinnitus [2,3]. In addition, upper airway obstruction caused by AH is a major contributor to obstructive sleep apnea, which commonly presents with snoring and mouth breathing. Prolonged obstructive sleep apnea may result in craniofacial abnormalities, often referred to as “adenoid facies.” Chronic hypoxia related to AH can negatively impact children’s development. These effects include poor concentration, learning difficulties, behavioral problems, and growth retardation [4]. Timely and proper management is crucial for the prevention and treatment of AH. For parents, access to reliable health education is key to early detection, timely diagnosis, appropriate treatment, and the prevention of complications.

As mobile internet use continues to grow, online platforms have become a major source of health information for parents, especially in China. Parents and guardians now frequently turn to online channels and social media to learn about common childhood illnesses and related health guidance [5,6]. Nevertheless, such online health information varies greatly in overall quality, and misinformation still poses a prominent problem. Scholars have already explored the quality of online health information related to AH and other related pediatric conditions. For instance, Jiang et al [7] noted significant differences in the quality, readability, and reliability of AH-related information across Chinese and English websites. Their findings also revealed obvious shortages of accessible and accurate public health education resources. Although online health information is easily accessible nowadays, many parents still have knowledge gaps and misconceptions. Such misunderstandings can easily mislead parents and influence their decisions regarding medical consultation and subsequent treatment.

Issues surrounding the quality and reliability of online health information are not confined to Chinese platforms; they are a global phenomenon. Research on health-related video content across international platforms has yielded similar findings [8]. In one investigation of AH-themed videos on YouTube, researchers noted large differences in content quality and reliability. A considerable number of videos did not provide complete, evidence-based explanations. Collectively, existing literature confirms uneven information quality and prevalent misinformation throughout online health media environments.

Short-video platforms, defined as digital platforms that disseminate brief, user-generated audiovisual content optimized for mobile viewing and algorithm-driven distribution, have grown into major channels for sharing health information with the public. Through concise, vivid, and easy-to-understand forms, these platforms can convey complicated medical knowledge clearly. This enables users to learn health-related content during their daily spare time and other fragmented periods throughout the day [9,10]. In China, Bilibili (Bilibili Inc), TikTok (ByteDance Inc), and Xiaohongshu (Xingyin Information Technology Co Ltd) are all highly popular online platforms. They now deeply influence how ordinary people acquire and access health information.

According to 2023 industry statistics, Bilibili has approximately 368 million monthly active users [11]. Douyin, China’s local counterpart of TikTok, has surpassed 1 billion overall users, with average daily use exceeding 1.5 hours [12]. Xiaohongshu reaches approximately 300 million monthly active users, where female users account for 72% of the total user base [13]. On these platforms, users can easily locate AH and other health-related videos through simple keyword searches, with no mandatory account registration or payment required.

There is no unified standardized regulation or professional peer-review system for such video resources, resulting in large quality gaps in completeness, understandability, actionability, reliability, and overall quality. Much of the existing video content is of low quality, and some content contains inaccurate information that may affect the public’s adoption of proper health behaviors [14,15]. In the fields of otolaryngology and head and neck surgery, previous studies have evaluated short-form video content on topics such as pediatric tonsillectomy [16], cholesteatoma [17], tinnitus [18], nasopharyngeal carcinoma [19], laryngeal cancer [20], thyroid nodules [21], and thyroid cancer [22-24]. All these studies indicate that such online content is often not of ideal quality and may contain misinformation. This underscores the importance of greater involvement of health care professionals in improving the quality of online health content [25,26].

Although some related studies are available, most existing work concentrates on conventional text-based websites or on other disease topics. Little research has focused specifically on AH short-video content hosted on Chinese online platforms. Given the widespread popularity and growing influence of short-video platforms among parents, evaluating the quality of AH-related content on these platforms is essential.

This study aimed to assess the completeness, understandability, actionability, reliability, and overall quality of AH-related videos on Bilibili, TikTok, and Xiaohongshu and to explore factors associated with quality metrics, including uploader type, video characteristics, and user engagement, thereby addressing this important gap in the Chinese short-video literature.

## Methods

### Search Strategy and Data Collection

This study applied the Chinese keyword “腺样体肥大” (“adenoid hypertrophy”) to screen relevant videos from Bilibili, TikTok, and Xiaohongshu. We included the top 100 videos sorted by overall comprehensive relevance. The 3 platforms were chosen on practical grounds. All 3 platforms are highly popular within China’s short-video market, although each has a distinct content orientation. Bilibili prioritizes knowledge-sharing posts, TikTok delivers content mainly through algorithmic recommendation, whereas Xiaohongshu centers on lifestyle-based user-generated content. We limited the sample to the top 100 videos from each platform ranked by comprehensive relevance, as prior research suggests that users rarely browse beyond the first several pages of search results and are most influenced by highly ranked content [14,27]. To minimize potential bias introduced by platform-specific recommendation algorithms, newly created accounts were used to perform all searches. The search was conducted between April 8, 2025, and April 15, 2025, and included all videos available on each platform at the time of retrieval, regardless of upload date. Duplicate videos, silent videos, advertisements, and content unrelated to the topic were excluded. Videos presented in multiple parts were treated as a single entry. For each included video, the following characteristics were recorded and analyzed: source platform; video duration; number of likes, collections, comments, and shares; uploader identity; verification status of the uploader (as indicated by the platform); and video production style. Two reviewers independently screened all retrieved videos based on predefined inclusion and exclusion criteria. Discrepancies were resolved through discussion; if consensus could not be reached, a third reviewer adjudicated the final decision.

### Assessment of Video Content and Quality

We evaluated the completeness, understandability, actionability, reliability, and overall quality of the included videos. On the basis of the 2025 Chinese Expert Consensus on Adenoid Hypertrophy [4] the 2023 German S2k Guideline for the Diagnosis and Treatment of Adenoid Hypertrophy [28], and previously published methods for video content analysis [29,30], our research team developed an integrity scoring framework. The framework was further refined through consultation with two otolaryngology professors (refer to the Acknowledgments section for details). The framework covered 6 domains: definition, symptoms and complications, risk factors, diagnosis, treatment or management, and outcomes. Each domain was scored on a 5-point scale (0‐2) using predefined criteria: 0=“no content,” 0.5=“minimal content,” 1=“some content,” 1.5=“most content,” and 2=“extensive content” (Multimedia Appendix 1).

Understandability and actionability were assessed using the Patient Education Materials Assessment Tool for Audiovisual Materials (PEMAT-A/V) [31]. This instrument includes 17 items, with 13 items evaluating understandability and 4 items assessing actionability. Each item was rated as “agree” (1 point), “disagree” (0 points), or “not applicable.” Scores for understandability (Patient Education Materials Assessment Tool–Understandability [PEMAT-U]) and actionability (Patient Education Materials Assessment Tool–Actionability [PEMAT-A]) were calculated as follows: (total score÷total possible score)×100, with higher scores indicating better performance (Multimedia Appendix 2).

A modified version of DISCERN (mDISCERN) instrument was used to assess the reliability of the video content. The mDISCERN is a well-established and widely used tool for evaluating the quality of health information in both research and clinical settings [20,32]. Video reliability was evaluated based on 5 criteria: clarity, traceability, balance, relevance, and robustness. Each item was scored as 1 (“yes”) or 0 (“no”), yielding a total score ranging from 0 to 5, with higher scores indicating greater reliability (Multimedia Appendix 3).

The Global Quality Scale (GQS) was used to assess overall video quality. The GQS is commonly applied to evaluate the quality of online health information and uses a 5-point scale, where 1 indicates poor quality and 5 indicates excellent quality [33,34] (Multimedia Appendix 4). These instruments were selected to assess complementary dimensions of video quality. The PEMAT-A/V evaluates understandability and actionability from a patient education perspective, the mDISCERN assesses the reliability and transparency of health information, and the GQS provides an overall global assessment of educational quality.

Prior to formal assessment, the 2 primary reviewers (CH and YZ) jointly evaluated the first 20 videos to calibrate scoring criteria. Both are registered nurses with 8 years of otorhinolaryngology experience and have received training in all assessment instruments used in this study (content completeness scoring, PEMAT-A/V, mDISCERN, and GQS). A third nurse (WJ), a head nurse with 30 years of experience, resolved any disagreements. All videos were independently evaluated by 2 reviewers. During this process, any statements that contradicted established clinical guidelines were recorded verbatim and thematically categorized as misleading (Multimedia Appendix 5). Discrepancies were initially resolved through discussion; if consensus could not be reached, a third reviewer adjudicated the final score. Interrater agreement was assessed using Cohen kappa, interpreted according to the Landis and Koch criteria [35]. The κ values ranged from 0.77 to 0.993 across all measures: content completeness (κ=0.835-0.923), PEMAT-U (κ=0.789-0.993), PEMAT-A (κ=0.933-0.975), mDISCERN (κ=0.77-0.961), and GQS (κ=0.873). These results indicate acceptable-to-excellent interrater agreement.

Uploader verification status (“certification”) refers to platform-designated credentials indicating that an account has undergone official verification, typically confirming its affiliation with a licensed health care professional, medical institution, or recognized organization.

### Statistical Analysis

Statistical analyses and data visualization were performed using SPSS (version 26; IBM Corp) and R (version 4.5.3; R Foundation for Statistical Computing). Continuous variables were assessed for normality; normally distributed data are presented as mean (SD), whereas nonnormally distributed data are presented as median (IQR). Categorical variables are presented as frequencies and percentages. For comparisons between 2 independent groups (eg, medical vs nonmedical uploaders), the Mann-Whitney *U* test was used for continuous variables and the chi-square test was used for categorical variables. For comparisons among the 3 platforms (Bilibili, TikTok, and Xiaohongshu), the Kruskal-Wallis *H* test was applied. When significant differences were identified, post hoc pairwise comparisons were performed using Mann-Whitney *U* tests with Bonferroni correction. Spearman rank correlation analysis was used to examine associations between video characteristics (duration and engagement metrics, including likes, comments, shares, and collections) and quality-related scores (content completeness, reliability, PEMAT-U, PEMAT-A, and GQS). To identify independent predictors of overall video quality (GQS), a stepwise linear regression analysis was performed with candidate variables including video duration, log-transformed engagement metrics, uploader type, platform (dummy-coded with TikTok as the reference), PEMAT-U, and PEMAT-A. A 2-tailed *P*<.05 was considered statistically significant.

### Ethical Considerations

This study used only publicly available videos from Bilibili, TikTok, and Xiaohongshu. No personally identifiable information was collected, and there was no direct interaction with platform users. According to Article 32 of the Measures for Ethical Review of Life Science and Medical Research Involving Human Subjects (the official Chinese policy document measures for ethical review of life science and medical research involving human participants, Guowei Kejiao Fa, 2023, No 4) [36], research that uses lawfully obtained publicly available data without harming human subjects or involving sensitive personal information or commercial interests may be exempt from formal ethics review. Therefore, ethics approval was not sought for this study.

## Results

### Video Characteristics

**Figure 1.**
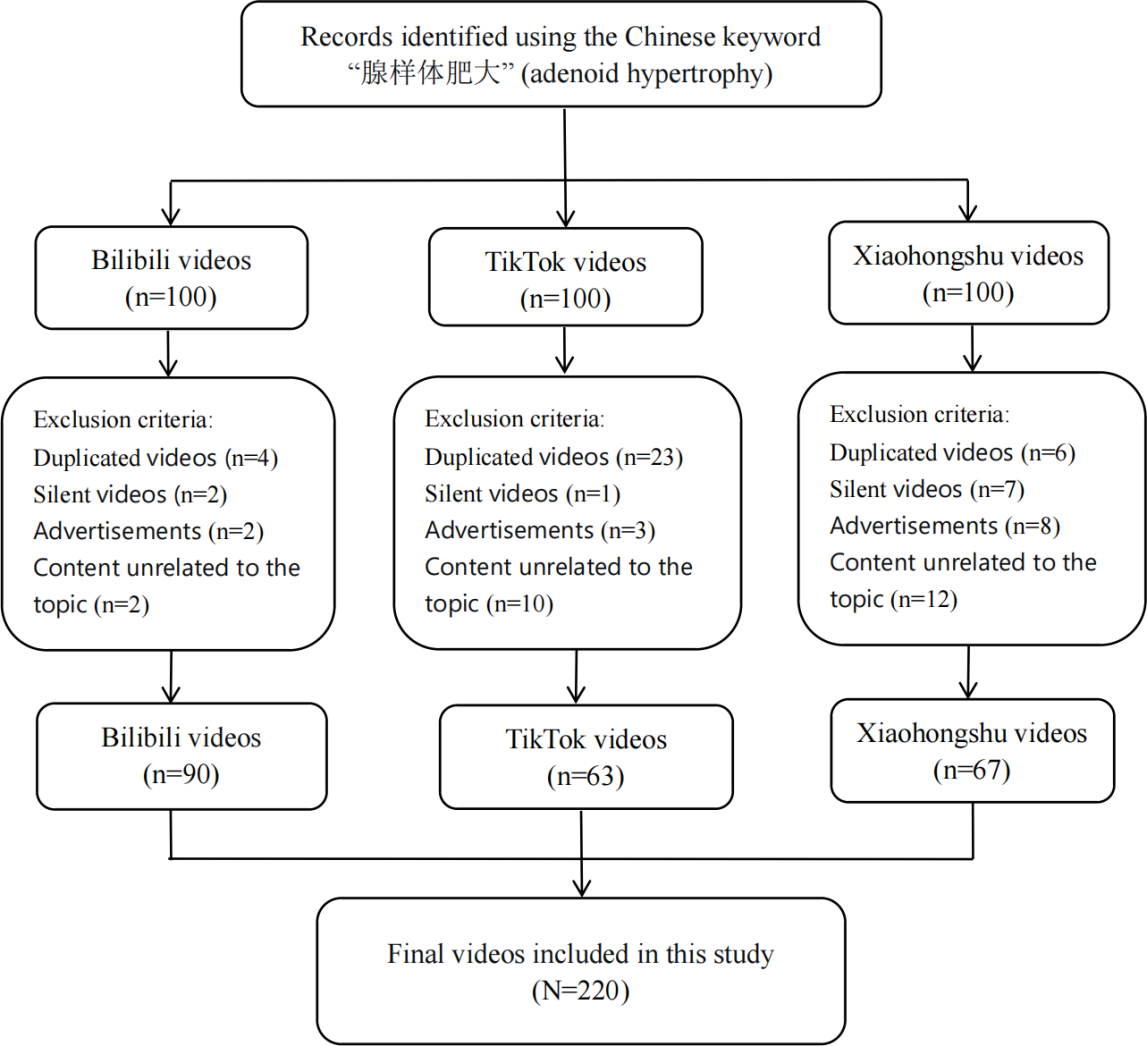
Flowchart of study selection.

As shown in Figure 1, a total of 220 videos on AH were included based on the predefined inclusion and exclusion criteria. Of these, 90 (40.9%) videos were from Bilibili, 63 (28.6%) from TikTok, and 67 (30.5%) from Xiaohongshu. Videos on Bilibili were significantly longer than those on TikTok and Xiaohongshu (*P*<.001). Xiaohongshu videos had significantly higher numbers of likes, collections, and comments than videos on Bilibili and TikTok (all *P*<.001), whereas no significant difference in the number of shares was observed among the 3 platforms (*P*=.09). Detailed characteristics are shown in Table 1.

**Table 1.** Characteristics of 220 adenoid hypertrophy–related videos from Bilibili, TikTok, and Xiaohongshu (China; April 8-15, 2025).

Variables	Total (N=220), median (IQR)	Bilibili (n=90), median (IQR)	TikTok (n=63), median (IQR)	Xiaohongshu (n=67), median (IQR)	*P* value
Video duration (seconds)	77.5 (49.0-162.0)	113.5 (66.5‐271.5)	73.0 (44.0-100.0)	63.0 (41.0‐127.5)	<.001
Likes	153.5 (35.2-717.0)	70.0 (11.2-291.0)	162.0 (63.0-1120.0)	498.0 (65.0-2456.0)	<.001
Collections	160.0 (29.5-715.5)	113.5 (17.5-356.5)	77.0 (29.5-677.0)	593.0 (73.5-2967.0)	<.001
Comments	20.0 (3.0-106.7)	6.5 (0-40.5)	29.0 (4.0-152.5)	52.0 (10.0-123.5)	<.001
Shares	136.5 (18.5-662.0)	113.5 (13.2-380.0)	84.0 (17.0-1005.0)	281.0 (33.0-1004.5)	.09

### Sources and Shooting Styles of Videos

The source and production characteristics of the videos differed significantly across platforms (all *P*<.001; Table 2). Medical professionals accounted for most videos on Bilibili (n=69, 76.7%) and TikTok (n=60, 95.2%), but for less than half of those on Xiaohongshu (n=29, 43.3%). Verification rates were high on Bilibili (n=90, 100%) and TikTok (n=60, 95.2%), but low on Xiaohongshu (n=30, 44.8%). Solo narration was the most common format across platforms (n=126, 57.3%). Xiaohongshu had a markedly higher proportion of animation- or action-based videos (n=22, 32.8%) than the other 2 platforms (n=5, ≤5.6%).

**Table 2.** Sources and production characteristics of videos on adenoid hypertrophy across 3 Chinese short-video platforms (N=220)^a^.

Variables	Total	Bilibili (n=90)	TikTok (n=63)	Xiaohongshu (n=67)	*P* value
Uploader type, n (%)					<.001
Medical professionals	158 (71.82)	69 (76.67)	60 (95.24)	29 (43.28)	
Nonmedical professionals	62 (28.18)	21 (23.33)	3 (4.76)	38 (56.72)	
Uploader identity, n (%)					<.001
Medical institutions	3 (1.36)	2 (2.22)	0 (0)	1 (1.49)	
Other related specialist physicians	31 (14.09)	24 (26.67)	2 (3.17)	5 (7.46)	
Otorhinolaryngologists	43 (19.55)	15 (16.67)	18 (28.57)	10 (14.93)	
Pediatricians	84 (38.18)	30 (33.33)	40 (63.49)	14 (20.9)	
Science popularization bloggers	28 (12.73)	15 (16.67)	0 (0.00)	13 (19.4)	
Parents of children with adenoid hypertrophy	31 (14.09)	4 (4.44)	3 (4.76)	24 (35.82)	
Certification status, n (%)					<.001
Certified	180 (81.82)	90 (100)	60 (95.24)	30 (44.78)	
Uncertified	40 (18.18)	0 (0)	3 (4.76)	37 (55.22)	
Video shooting style, n (%)					<.001^b^
Animation or action	27 (12.27)	5 (5.56)	0 (0.00)	22 (32.84)	
Medical scenes	49 (22.27)	15 (16.67)	23 (36.51)	11 (16.42)	
Other types	7 (3.18)	4 (4.44)	0 (0)	3 (4.48)	
PowerPoint presentation or course	4 (1.82)	4 (4.44)	0 (0)	0 (0)	
Question and answer	4 (1.82)	4 (4.44)	0 (0)	0 (0)	
Solo narration	126 (57.27)	57 (63.33)	39 (61.9)	30 (44.78)	
Television program or documentary	3 (1.36)	1 (1.11)	1 (1.59)	1 (1.49)	

a Fisher exact test was applied to uploader type, uploader identity, and certification status.

b Simulated *P* values.

### Video Content

**Figure 2.**
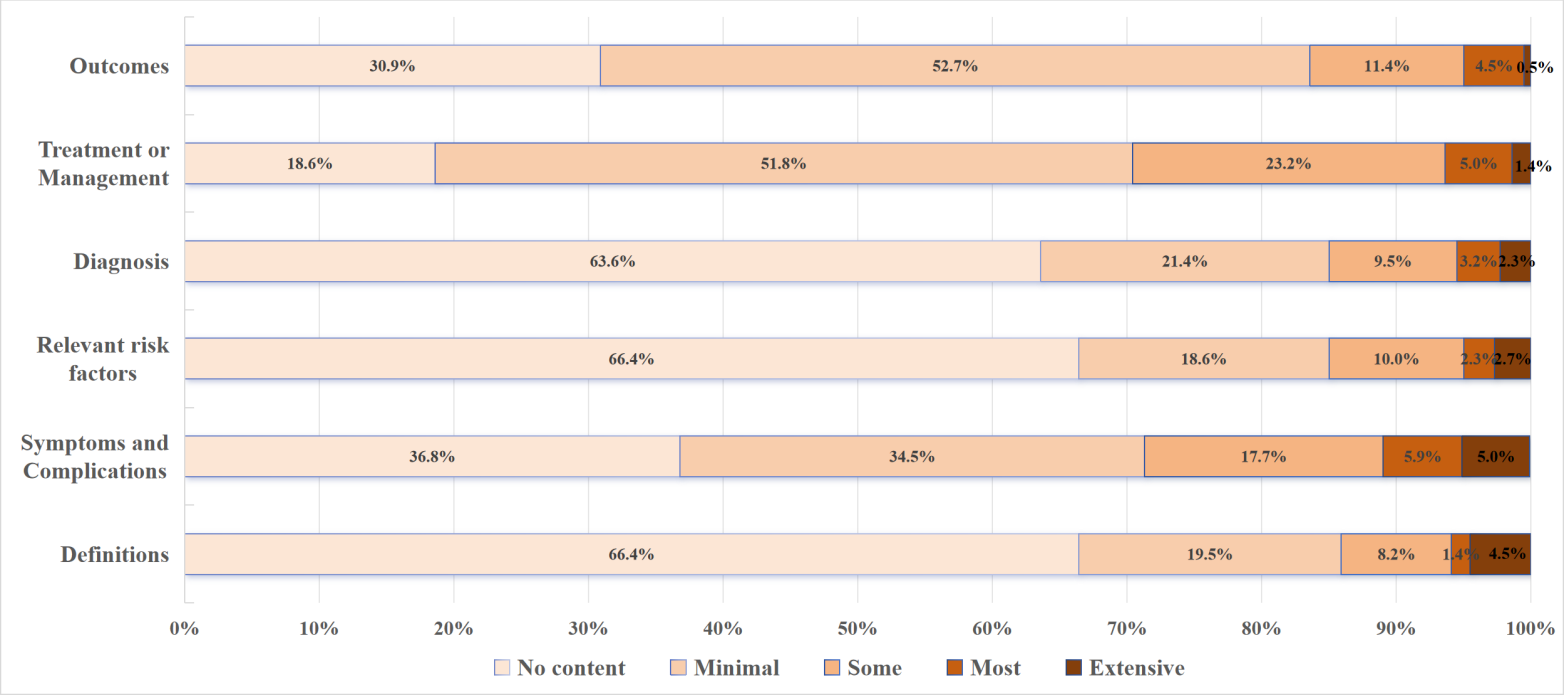
Percentage distribution of content completeness across 6 domains in adenoid hypertrophy–related videos.

As shown in Figure 2, treatment or management and symptoms and complications were the most frequently addressed topics, with 29.6% (n=65) and 28.6% (n=63) of videos, respectively, providing at least some relevant information. In contrast, definitions, risk factors, diagnostic approaches, and outcomes were rarely covered.

We further evaluated content completeness across platforms and uploader types. Completeness scores were generally low (median total score of 1.5-2 out of 12). As shown in Table 3, overall Kruskal-Wallis *H* tests revealed no significant differences for definitions or symptoms (both *P*>.05), but significant differences for risk factors (*H*=8.00; *P*=.018), diagnosis (*H*=8.99; *P*=.01), treatment (*H*=9.09; *P*=.011), outcomes (*H*=25.40; *P*<.001), and total completeness (*H*=18.52; *P*<.001). Post hoc comparisons with Bonferroni correction showed that Bilibili outperformed TikTok in risk factors (adjusted *P*=.028), diagnosis (adjusted *P*=.011), and outcomes (adjusted *P*=.001) and outperformed Xiaohongshu in treatment (adjusted *P*=.014) and outcomes (adjusted *P*<.001). Bilibili also had higher total completeness than both TikTok (adjusted *P*=.002) and Xiaohongshu (adjusted *P*=.001). No significant differences were observed between medical and nonmedical uploaders for any completeness domain (all *P*>.05).

**Table 3.** Content completeness scores across platforms and pairwise comparisons.

Content domains	Bilibili (n=90), median (IQR)	TikTok (n=63), median (IQR)	Xiaohongshu (n=67), median (IQR)	*P* value	Pairwise comparisons, Bonferroni-adjusted *P* values		
					Bilibili vs TikTok	Bilibili vs Xiaohongshu	TikTok vs Xiaohongshu
Definitions	0 (0-0.5)	0 (0‐0.5)	0 (0‐0)	.14	—^a^	—	—
Symptoms and complications	0.5 (0‐1.0)	0.5 (0‐1.0)	0.5 (0.5‐0.5)	.38	—	—	—
Risk factors	0 (0‐0.5)	0 (0‐0.5)	0 (0‐0)	.02	.028	.106	>.99
Diagnosis	0.5 (0.5‐0.5)	0 (0‐0.5)	0 (0‐0)	.01	.011	.171	>.99
Treatment or management	0.5 (0.5‐1.0)	0.5 (0.5‐0.5)	0.5 (0.5‐0.5)	.01	.105	.014	>.99
Outcomes	0.5 (0‐0.5)	0.5 (0‐0.5)	0 (0‐0)	<.001	.001	<.001	.781
Total score (range 0‐12)	2.0 (1.5‐4.0)	1.5 (0.5‐2.0)	1.5 (0.5‐2.8)	<.001	.002	.001	>.99

a Pairwise comparisons not performed because overall *P*>.05.

In addition, we identified misleading or non–evidence-based information in some videos (Multimedia Appendix 4). A total of 9 videos were clearly misleading. Examples included recommendations of *tuina*, dietary therapy, or herbal remedies as substitutes for surgery; claims that “90% obstruction can be cured by Chinese herbs”; and exaggerated statements about the consequences of not undergoing surgery (eg, “surgery must be performed immediately, otherwise growth and intelligence will be affected”). It should be noted that observations regarding specific content characteristics were derived from qualitative review, rather than from formal quantitative comparisons. In our dataset, most misleading videos were uploaded by nonprofessional creators. Among the 9 videos that remained accessible, 5 (55.5%) were uploaded by medical professionals (pediatricians or otolaryngologists).

### Comparison of Video Completeness and Quality Across Platforms

**Figure 3.**
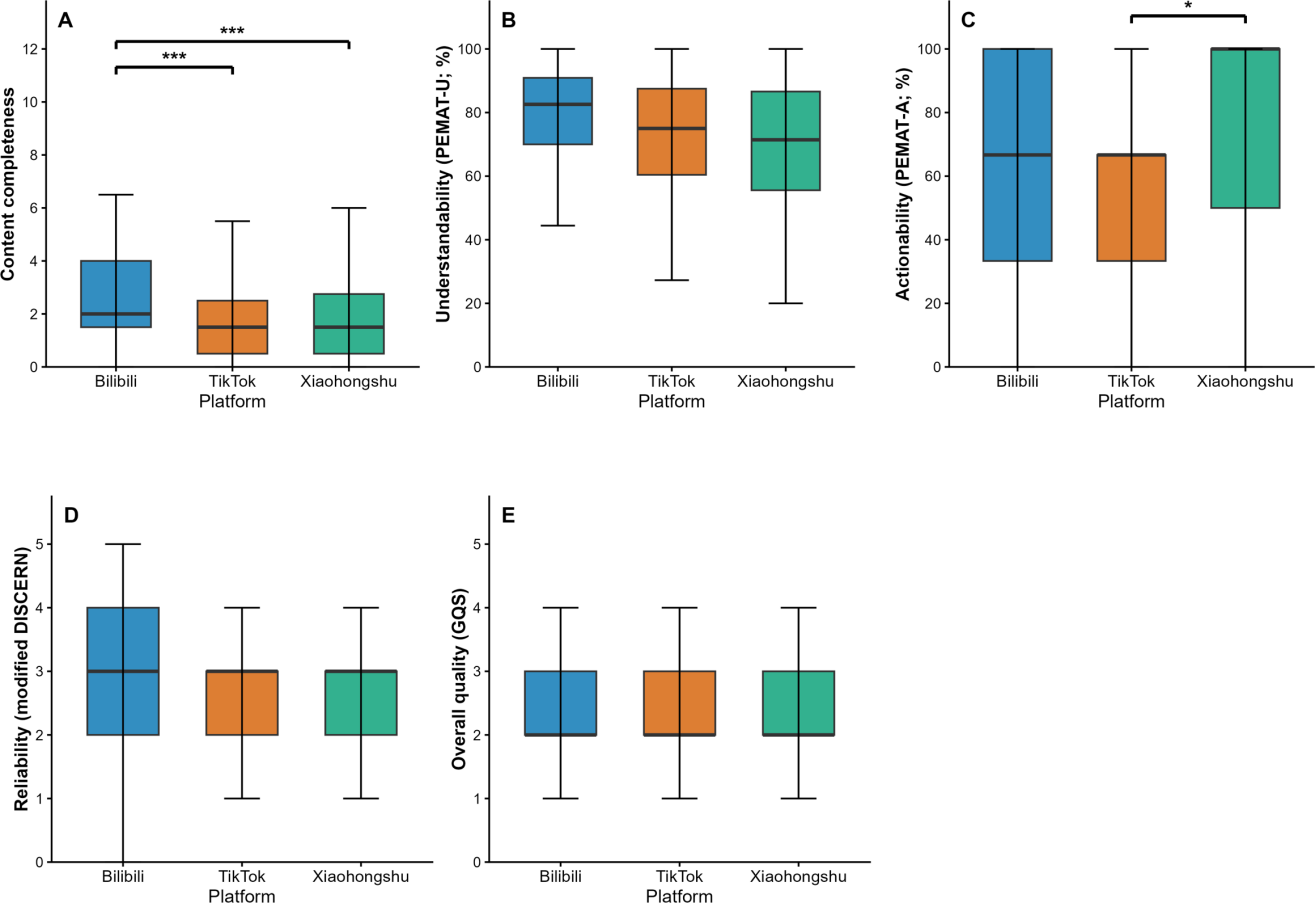
Comparison of video quality metrics across Bilibili, TikTok, and Xiaohongshu platforms in a cross-sectional analysis of 220 adenoid hypertrophy–related videos collected in China between April 8, 2025, and April 15, 2025. (A) Content completeness, (B) understandability (Patient Education Materials Assessment Tool–Understandability [PEMAT-U]), (C) actionability (Patient Education Materials Assessment Tool–Actionability [PEMAT-A]), (D) reliability (modified DISCERN), and (E) overall quality (Global Quality Scale [GQS]).*P<.05; **P<.01; ***P<.001.

As shown in Table 4, all 5 quality indicators differed significantly across platforms (all *P*<.05). For content completeness, post hoc comparisons with Bonferroni correction showed that Bilibili outperformed both TikTok (adjusted *P*<.001) and Xiaohongshu (adjusted *P*<.001). For actionability (PEMAT-A), Xiaohongshu scored higher than TikTok (adjusted *P*=.011). No other pairwise comparisons reached statistical significance. The median GQS score was 2 across platforms, with a significant but small difference (*P*=.041). Figure 3 provides a visual comparison.

**Table 4.** Comparison of video completeness and quality across platforms.

Variables	Total (N=220), median (IQR)	Bilibili (n=90), median (IQR)	TikTok (n=63), median (IQR)	Xiaohongshu (n=67), median (IQR)	*P* value
Content completeness	2.0 (1.0-3.0)	2.0 (1.5-4.0)	1.5 (0.5-2.0)	1.5 (0.5-2.8)	<.001
Patient Education Materials Assessment Tool–Understandability (%)	77.78 (62.50-89.72)	82.58 (70.00-90.91)	75 (60.42-87.50)	71.43 (55.56-86.61)	.027
Patient Education Materials Assessment Tool–Actionability (%)	66.67 (33.33-100.00)	66.67 (33.33-100.00)	66.67 (33.33-66.67)	100.00 (50.00-100.00)	.016
Modified DISCERN score	3 (2-3)	3 (2-4)	3 (2-3)	3 (2-3)	.028
Global Quality Scale score	2 (2-3)	2 (2-3)	2 (2-3)	2 (2-3)	.041

### Comparison by Uploader Type

**Figure 4.**
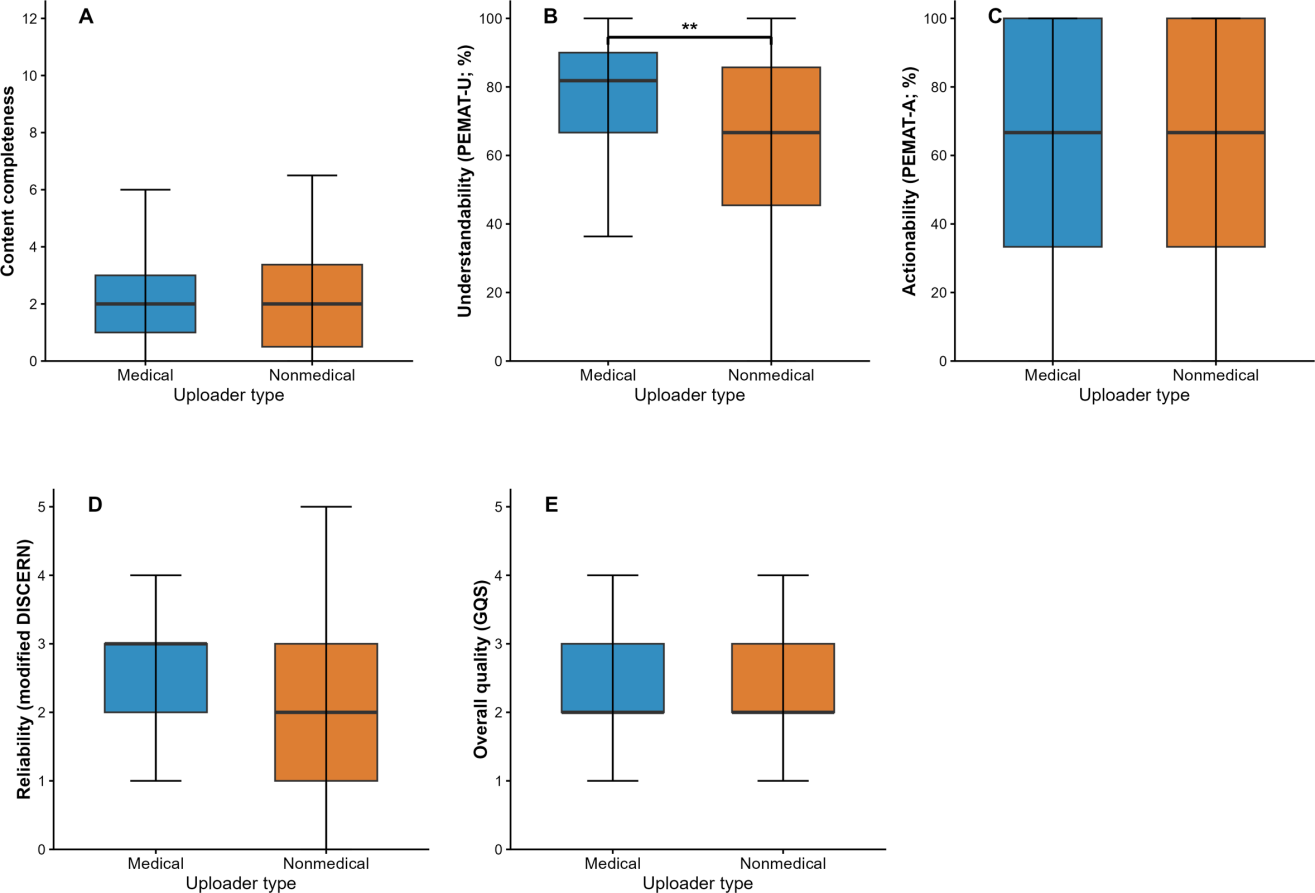
Comparison of video quality metrics by uploader type (medical vs nonmedical) in a cross-sectional analysis of 220 adenoid hypertrophy–related videos from Chinese short-video platforms collected between April 8, 2025, and April 15, 2025. (A) Content completeness, (B) understandability (Patient Education Materials Assessment Tool–Understandability [PEMAT-U]), (C) actionability (Patient Education Materials Assessment Tool–Actionability [PEMAT-A]), (D) reliability (modified DISCERN), and (E) overall quality (Global Quality Scale [GQS]). “Medical” denotes medical professionals, and “Nonmedical” denotes nonmedical professionals. *P<.05; **P<.01; ***P<.001.

As shown in Figure 4, videos uploaded by medical professionals had significantly higher understandability scores than those uploaded by nonmedical individuals (*Z*=−3.22; *P* =.001). No significant differences were found for completeness, actionability, reliability, or overall quality (all *P*>.05).

### Correlation Analysis

**Figure 5.**
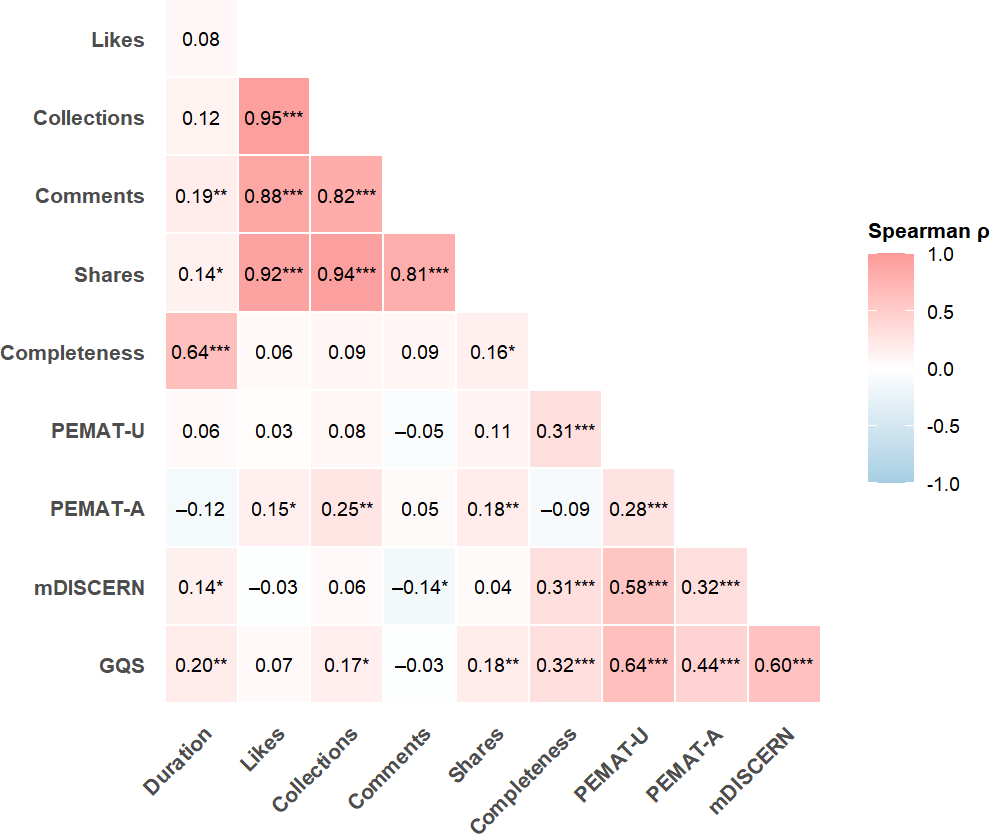
Spearman correlation heat map of video characteristics (duration, likes, collections, comments, and shares), content completeness, and quality metrics (Patient Education Materials Assessment Tool–Understandability [PEMAT-U], Patient Education Materials Assessment Tool–Actionability [PEMAT-A], modified version of DISCERN [mDISCERN], and Global Quality Scale [GQS]). Only the lower triangular part of the matrix is shown. Colors range from blue (negative correlation) to red (positive correlation). *P<.05; **P<.01; ***P<.001.

Spearman correlation analysis (Figure 5) showed that video duration was strongly positively correlated with content completeness (ρ=0.64; *P*<.001), but not with other quality indicators (all ρ≤0.2). Weak but statistically significant correlations were observed between shares and both completeness (ρ=0.16; *P*=.02) and actionability (ρ=0.18; *P*=.006). As shown in the heat map, engagement metrics (likes, collections, comments, and shares) were highly correlated with each other (ρ>0.80; *P*<.001), and understandability (PEMAT-U) was strongly associated with reliability (mDISCERN) and overall quality (GQS; ρ=0.58 and 0.64, respectively; *P*<.001). No other meaningful associations were found.

### Stepwise Linear Regression Analysis

Stepwise linear regression analysis was performed to identify independent predictors of overall video quality (GQS; Table 5). Candidate variables included PEMAT-U, PEMAT-A, video duration (seconds), log-transformed engagement metrics (likes, shares, comments, and collections), uploader type (medical professional vs nonmedical professional), and platform (dummy-coded with TikTok as the reference). The stepwise procedure (entry criterion *P*≤.05; removal criterion *P*≥.10) yielded 4 models. The final model (model 4) was statistically significant (adjusted *R*^2^=0.461; *F*_4,215_=47.91; *P*<.001) and included 4 predictors: PEMAT-U (β=.573; *P*<.001), PEMAT-A (β=.230; *P*<.001), video duration (β=.166; *P*=.001), and uploader type (β=.148; *P*=.005). No other variables entered the model. These results indicate that understandability was the strongest predictor of perceived video quality, followed by actionability and video length, while uploader type had a smaller but significant effect. Engagement metrics and platform did not contribute independently after accounting for content-related characteristics.

**Table 5.** Stepwise linear regression predictors of overall video quality (Global Quality Scale, final model)^a^.

Predictors	β (95% CI)
Patient Education Materials Assessment Tool–Understandability	0.573 (0.021‐0.030)
Patient Education Materials Assessment Tool–Actionability	0.230 (0.003‐0.009)
Video duration (seconds)	0.166 (0.000‐0.001)
Uploader type (nonmedical vs medical)	0.148 (0.086‐0.486)

a The final model was statistically significant (adjusted *R*2=0.461; *F*_4,215_=47.91; *P*<.001). The constant term was omitted. Intermediate models (models 1-3) are available in Multimedia Appendix 6.

## Discussion

### Principal Findings

This study provides a systematic evaluation of AH-related videos across 3 major Chinese short-video platforms using multiple validated instruments. Overall video quality was suboptimal, with substantial variability across platforms. Bilibili showed a significant advantage only in content completeness; for understandability and reliability, overall differences were observed, but pairwise comparisons did not reach statistical significance after Bonferroni correction. Xiaohongshu had higher actionability than TikTok, yet no platform performed well across all dimensions. Most engagement metrics were unrelated to content quality, except for weak positive correlations between shares and completeness and actionability. Stepwise linear regression further identified understandability (PEMAT-U) as the strongest independent predictor of overall quality (GQS), followed by actionability (PEMAT-A), video duration, and uploader type; engagement metrics and platform did not enter the model. Notably, a substantial proportion of misleading videos originated from medical professionals, in contrast to the pattern observed in previous YouTube-based research [8]. These findings collectively highlight the critical role of content clarity and actionability, the limitations of engagement metrics as quality proxies, and the need for better content oversight regardless of uploader credentials.

### Stepwise Regression Predictors

Stepwise regression identified understandability (PEMAT-U) as the strongest independent predictor of overall quality (GQS), followed by actionability (PEMAT-A), video duration, and uploader type. Engagement metrics and platform did not enter the final model, further confirming that popularity and platform differences are unreliable proxies for content quality.

This finding aligns with 2 recent studies. A study on adolescent sexuality education also found PEMAT-U to be the strongest positive predictor of GQS (β=.485), surpassing actionability, video duration, and uploader type [37]. Similarly, an analysis of uterine fibroid videos reported that the “completeness score,” “source,” and “PEMAT scores” were significant predictors of video quality, confirming that content characteristics outweigh simple popularity or uploader identity in determining educational value [38].

Our finding that engagement metrics were not significant predictors aligns with the “credibility-evidence gap” documented in a broad evaluation of physician-generated health claims [39]. In that study, 62.5% of medical claims rested on “very low” or “no evidence,” yet low-evidence videos received 34.6% more views than high-evidence ones, directly mirroring our observation that engagement metrics do not predict quality. This evidence suggests that current algorithm recommendations, which place substantial weight on user engagement, may unintentionally prioritize low-quality content. Consequently, platform algorithms should be adjusted to promote comprehensible, actionable content rather than simply rewarding videos that generate high volumes of likes, comments, or shares.

### Sources of Misleading Information

Misinformation has been reported in health-related videos on platforms such as YouTube and TikTok across various conditions, including atopic dermatitis, ophthalmology, and oral health [14,15,40]. Similarly, a YouTube-based study on AH found that 21.5% of videos were misleading, with the vast majority uploaded by nonprofessional sources [8].

Consistent with prior reports [8], most misleading videos in our dataset came from nonprofessional creators. However, even verified professional accounts can occasionally produce misleading content. One possible explanation is that some clinicians use simplified or absolute language to increase engagement or that traditional Chinese medicine promotions sometimes blur the boundary between evidence-based and unverified claims. Thus, misleading information can originate from both nonprofessional and professional sources, and because viewers trust verified accounts, misinformation from the latter is especially concerning.

### Practical Implications

Short-video platforms have become a common source of health information for parents of children with conditions such as AH. Our findings indicate that content quality is generally low, and neither professional credentials nor engagement metrics reliably predict actual quality. In fact, understandability was the strongest independent predictor of overall quality, and misleading videos appeared from both nonprofessional and verified professional sources.

These results point to several actionable directions. For clinicians and health educators, improving the clarity and actionability of video content should take priority over simply displaying professional titles. Oversimplified or absolute statements—even from experts—can be misleading. For platform developers, the weak correlation between engagement metrics and quality suggests that ranking algorithms based heavily on likes, shares, or comments may inadvertently promote low-quality content. Incorporating evidence-based indicators such as content completeness and understandability into ranking systems would be a more effective strategy. For regulators, account verification alone is insufficient. Content-based review mechanisms are essential to counter misinformation, regardless of whether the uploader is a medical professional.

### Limitations and Future Directions

This study has several limitations. First, it only included Chinese short videos related to AH on Bilibili, TikTok, and Xiaohongshu. This leads to selection bias, which limits the external validity of the results and makes them unrepresentative of content quality on other platforms and in different cultural contexts. Second, although 4 validated scales were used in the study, the inherent flaws and systematic biases of these scales cannot be completely avoided. Third, this study was a cross-sectional survey conducted at a specific time point. As online short-video content is frequently updated, edited, and deleted, the research conclusions may lack long-term stability. Future research can expand the sampling scope to cover more platforms and multilingual groups. It may also be feasible to adopt automated content analysis methods and longitudinal research designs to further explore the impact of online health information on users’ health cognition, behavioral habits, and long-term health outcomes.

### Conclusions

This study evaluated the overall quality of educational videos related to AH on Bilibili, TikTok, and Xiaohongshu. The results showed that the overall quality of such short videos was relatively low. Videos on Bilibili had higher content completeness, while Xiaohongshu performed better than TikTok in actionability. Interaction metrics (likes, shares, and comments) exhibited weak or even no correlation with video content quality. This implies that current platform algorithms may inadvertently promote the spread of low-quality educational content. Stepwise regression analysis indicated that understandability (PEMAT-U) was the strongest predictor of overall video quality, followed by actionability, video duration, and uploader type. These findings suggest that easy-to-understand content with practical guiding value matters more than creators’ professional qualifications or video length. Misleading information was identified in videos released by both nonprofessional creators and medical professionals. Simple account verification alone cannot ensure the authenticity and reliability of online educational content. On the basis of the findings, medical practitioners are advised to produce popular science content that is easy to understand and practically informative, short-video platforms should optimize recommendation algorithms that overly depend on interaction data, and relevant regulatory departments should improve the review and supervision systems focused on content quality.

## Supplementary material

10.2196/82923 Multimedia Appendix 1 Adenoid hypertrophy video content completeness scoring framework.

10.2196/82923 Multimedia Appendix 2 Patient Education Materials Assessment Tools.

10.2196/82923 Multimedia Appendix 3 Description of the modified DISCERN score.

10.2196/82923 Multimedia Appendix 4 Description of the Global Quality Scale.

10.2196/82923 Multimedia Appendix 5 Representative examples of video content themes and potentially misleading or non−evidence-based claims identified during content analysis.

10.2196/82923 Multimedia Appendix 6 Stepwise linear regression intermediate models (models 1-3) for Global Quality Scale prediction (N=220).

## Abbreviations

AH: adenoid hypertrophy
GQS: Global Quality Scale
mDISCERN: modified version of DISCERN
PEMAT-A: Patient Education Materials Assessment Tool–Actionability
PEMAT-A/V: Patient Education Materials Assessment Tool for Audiovisual Materials
PEMAT-U: Patient Education Materials Assessment Tool–Understandability

## References

[1] Pereira L, Monyror J, Almeida FT (2018). Prevalence of adenoid hypertrophy: a systematic review and meta-analysis. Sleep Med Rev.

[2] Hu R, Xia L, Shi C, Zhou Y, Guo X (2024). Otitis media with effusion in preschool children with adenoid hypertrophy: risk factors and nursing care. Nurs Open.

[3] Yang A, Jv M, Zhang J, Hu Y, Mi J, Hong H (2023). Analysis of risk factors for otitis media with effusion in children with adenoid hypertrophy. Risk Manag Healthc Policy.

[4] Children’s Otolaryngology Committee of the Pediatric Branch of the Chinese Medical Doctor Association, Pediatric Allergic Committee of Chinese Maternal and Child Health Association, Otolaryngology-Head and Neck Surgery Branch of Asia-Pacific Association of Medicine and Bio-Immunology (2025). Expert consensus on clinical diagnosis and treatment management of adenoid hypertrophy in children [Article in Chinese]. Chin J Pract Pediatr.

[5] Frey E, Bonfiglioli C, Brunner M, Frawley J (2022). Parents’ use of social media as a health information source for their children: a scoping review. Acad Pediatr.

[6] Mertens E, Ye G, Beuckels E, Hudders L (2024). Parenting information on social media: systematic literature review. JMIR Pediatr Parent.

[7] Jiang Z, Yang X, Chen F, Liu J (2023). Critical analysis and cross-comparison between English and Chinese websites providing online medical information for patients with adenoid hypertrophy: cross-sectional study. JMIR Form Res.

[8] Abeş D, Tuhanioğlu B, Demirtaş MS (2026). Assessment of the quality and reliability of YouTube videos on adenoid hypertrophy. Eur Arch Otorhinolaryngol.

[9] Feng B, Malloch YZ, Kravitz RL (2021). Assessing the effectiveness of a narrative-based patient education video for promoting opioid tapering. Patient Educ Couns.

[10] He Z, Wang Z, Song Y (2023). The reliability and quality of short videos as a source of dietary guidance for inflammatory bowel disease: cross-sectional study. J Med Internet Res.

[11] Bilibili user base and characteristics analysis (2025). Tencent.

[12] Douyin's monthly active users in China surpass 1 billion: the average person spends over 1.5 hours a day on Douyin. Sohu.

[13] (2025). 2025 “active users” research report (Xiaohongshu platform). https://www.sohu.com/a/888790524_121094725.

[14] Mueller SM, Hongler VN, Jungo P (2020). Fiction, falsehoods, and few facts: cross-sectional study on the content-related quality of atopic eczema-related videos on YouTube. J Med Internet Res.

[15] Sampige R, Rodgers EG, Huang A, Zhu D (2024). Education and misinformation: exploring ophthalmology content on TikTok. Ophthalmol Ther.

[16] Strychowsky JE, Nayan S, Farrokhyar F, MacLean J (2013). YouTube: a good source of information on pediatric tonsillectomy?. Int J Pediatr Otorhinolaryngol.

[17] Reddy R, Cheng H, Jufas N, Patel N (2023). Assessing the quality of patient information for cholesteatoma on the video sharing platform YouTube. Otol Neurotol.

[18] Huang C, Lan H, Jiang F, Huang Y, Lai D (2024). The quality and reliability of patient education regarding sound therapy videos for tinnitus on YouTube. PeerJ.

[19] Tan DJ, Ko TK, Fan KS (2023). The readability and quality of web-based patient information on nasopharyngeal carcinoma: quantitative content analysis. JMIR Form Res.

[20] Liu Z, Chen Y, Lin Y (2024). YouTube/ Bilibili/ TikTok videos as sources of medical information on laryngeal carcinoma: cross-sectional content analysis study. BMC Public Health.

[21] Chen Y, Wang Q, Huang X (2024). The quality and reliability of short videos about thyroid nodules on BiliBili and TikTok: cross-sectional study. Digit Health.

[22] Wang L, Li Y, Gu J, Xiao L (2023). A quality analysis of thyroid cancer videos available on TikTok. Front Public Health.

[23] Aydin MA, Akyol H (2020). Quality of information available on YouTube videos pertaining to thyroid cancer. J Cancer Educ.

[24] Yang S, Zhan J, Xu X (2023). Is TikTok a high-quality source of information on thyroid cancer?. Endocrine.

[25] Suarez-Lledo V, Alvarez-Galvez J (2021). Prevalence of health misinformation on social media: systematic review. J Med Internet Res.

[26] Stellefson M, Paige SR, Chaney BH, Chaney JD (2020). Evolving role of social media in health promotion: updated responsibilities for health education specialists. Int J Environ Res Public Health.

[27] Zheng S, Tong X, Wan D, Hu C, Hu Q, Ke Q (2023). Quality and reliability of liver cancer-related short Chinese videos on TikTok and Bilibili: cross-sectional content analysis study. J Med Internet Res.

[28] Ahmad Z, Krüger K, Lautermann J (2023). Adenoid hypertrophy-diagnosis and treatment: the new S2k guideline. HNO.

[29] Kong W, Song S, Zhao YC, Zhu Q, Sha L (2021). TikTok as a health information source: assessment of the quality of information in diabetes-related videos. J Med Internet Res.

[30] Zhang J, Yuan J, Zhang D (2024). Short video platforms as sources of health information about cervical cancer: a content and quality analysis. PLoS One.

[31] Shoemaker SJ, Wolf MS, Brach C (2014). Development of the Patient Education Materials Assessment Tool (PEMAT): a new measure of understandability and actionability for print and audiovisual patient information. Patient Educ Couns.

[32] Nie SJ, Ning SN, Ding QC, Hou J, Luo YY, Wang HY (2025). Quality analysis of stroke-related videos on video platforms: cross-sectional study. JMIR Form Res.

[33] Haddad F, Abou Shahla W, Saade D (2024). Investigating topical steroid withdrawal videos on TikTok: cross-sectional analysis of the top 100 videos. JMIR Form Res.

[34] Zhou X, Ma G, Su X (2025). The reliability and quality of short videos as health information of guidance for lymphedema: a cross-sectional study. Front Public Health.

[35] Landis JR, Koch GG (1977). The measurement of observer agreement for categorical data. Biometrics.

[36] (2023). Notice on issuing the measures for ethical review of life science and medical research involving human subjects. National Health Commission of the People's Republic of China.

[37] Wang L, Shu X, Huang J, Yan W, Zhao D (2025). Quality and reliability of adolescent sexuality education on Chinese video platforms: sentiment-topic analysis and cross-sectional study. JMIR Form Res.

[38] Wang L, Chen Y, Zhao D, Xu T, Hua F (2025). Quality and dissemination of uterine fibroid health information on TikTok and Bilibili: cross-sectional study. JMIR Form Res.

[39] Kang E, Lee H, Choi J, Ju H (2026). The quality of evidence of and engagement with video medical claims. JAMA Netw Open.

[40] Riyaz MM, Gousalya V, Anu V (2024). Oral health misinformation on YouTube - a content analysis. J Pharm Bioallied Sci.

