# Supplementary material for: Videos on Bilibili, TikTok, and Xiaohongshu as Sources of Medical Information on Adenoid Hypertrophy: Cross-Sectional Content Analysis
Source: JMIR Form Res. 2026 Jun 18;10:e82923. doi: 10.2196/82923 (PMC13278250; doi:10.2196/82923)
Supplement: Multimedia Appendix 1 [file formative-v10-e82923-s001.docx]

**Adenoid Hypertrophy Video Content Completeness Scoring Framework**

The development of this content completeness scoring framework is grounded in the “Chinese Expert Consensus on Clinical Diagnosis and Treatment Management of Adenoid Hypertrophy in Children” (Chinese Medical Doctor Association, 2025) [1] and the “German S2k Guideline: Adenoid Hypertrophy – Diagnosis and Treatment” (Ahmad et al., 2023) [2].

This was reviewed and agreed upon by two otorhinolaryngology experts with clinical experience in managing adenoid hypertrophy.

**1. Definition**

1. Anatomy and location of the adenoids (pharyngeal tonsil, part of Waldeyer's lymphatic ring)
2. Pathological enlargement criteria (vs. physiological hyperplasia)
3. Primary affected population: children, peak age 1–6 years
4. Epidemiology: global prevalence (about 34.5% in children)
5. Epidemiology: prevalence in China (common disease)
6. Physiological involution begins after age 6 years and completes by puberty
7. Association with recurrent upper respiratory infections and allergic rhinitis

**2. Symptoms and Hazards**

1. Nasal obstruction and chronic mouth breathing
2. Loud snoring, obstructive sleep apnea (OSA), and nocturnal enuresis
3. Otologic complications: hearing loss, recurrent otitis media with effusion (OME)
4. Eustachian tube dysfunction leading to cholesteatoma and speech delay
5. Craniofacial developmental risks: “adenoid facies” (long face, open mouth, dental malocclusion)
6. Neurocognitive/behavioral impacts: poor concentration, learning difficulties, growth retardation
7. Other complications: chronic rhinosinusitis, upper airway cough syndrome

**3. Relevant Risk Factors**

1. Recurrent upper respiratory infections
2. Allergic rhinitis
3. Chronic rhinosinusitis
4. Environmental irritants (e.g., tobacco smoke)
5. Gastroesophageal reflux
6. Obesity
7. Immunological factors (antigen exposure, allergy)

**4. Diagnosis**

1. Clinical history: nasal obstruction, apnea, snoring, sleep disturbance, hearing impairment
2. Visual inspection for adenoid facies (permanently open mouth, visible tongue tip)
3. Nasal endoscopic examination (gold standard; choanal obstruction ≥51%)
4. Lateral neck X-ray: adenoid/nasopharynx (A/N) ratio measurement (≥0.71 indicates pathological hypertrophy)
5. Polysomnography for suspected OSA (obstructive apnea-hypopnea index >1 event/h)
6. Audiometric testing (tympanometry, pure-tone audiometry) for middle ear effusion
7. Differential diagnosis: nasopharyngeal masses (juvenile angiofibroma, carcinoma, lymphoma, Thornwaldt cyst)
8. Diagnostic allergy tests for inhaled allergens
9. Preoperative coagulation assessment (standardized questionnaire)

**5. Treatment / Management**

**Conservative management**

Watchful waiting for mild/isolated cases

**Medical management**

1. Intranasal corticosteroids (off-label use, evidence of benefit)
2. Leukotriene receptor antagonists (montelukast)
3. Antihistamines (for allergic comorbidity)
4. Nasal saline irrigation
5. Immunomodulators (bacterial lysates, spleen aminopeptide)
6. Short-term topical decongestants (for severe obstruction)
7. Mucolytics, antibiotics (when infection is present)

**Surgical management (adenoidectomy)**

1. Indications: severe symptoms, failure of conservative treatment (≥1–3 months), OSA, recurrent otitis media, speech delay, dental malocclusion, cardiopulmonary complications (full list includes 10 specific indications)
2. Surgical techniques: conventional curettage (standard in Germany), microdebrider, low-temperature coblation
3. Outpatient vs. inpatient care; age considerations
4. Perioperative care, complication prevention, postoperative monitoring

**Adjuvant therapies**

Allergy control, continuous positive airway pressure (CPAP) for severe OSA

**6. Outcomes**

1. Symptom resolution rates after medical vs. surgical treatment
2. Complication risks: hemorrhage (primary ≤24h, secondary >24h; rate 0.5%–8.0%), velopharyngeal insufficiency, infection, tooth damage, Eustachian tube injury
3. Long-term developmental and cognitive outcomes after treatment
4. Prognostic factors: age at intervention (younger age higher recurrence), OSA severity, comorbidities, obesity
5. Recurrence rate and management of recurrent adenoid hypertrophy
6. Quality of life improvement after surgery

**Scoring Instructions**

Each domain is scored according to the following criteria:

0 points = no content (the domain is not mentioned at all)

0.5 points = minimal content (only one or two sub-items mentioned superficially)

1 point = some content (three sub-items mentioned, or a partial explanation)

1.5 points = most content (four or more sub-items mentioned, with reasonable detail)

2 points = extensive content (almost all sub-items mentioned, with detailed and accurate explanation)

Total completeness score ranges from 0 to 12 points.

**Example Scoring (for illustration)**

| Definition | Symptoms and Hazards | Relevant Risk Factors | Diagnosis | Treatment / Management | Outcomes | Total |
| --- | --- | --- | --- | --- | --- | --- |
| 1.5 | 1.0 | 0.5 | 1.5 | 2.0 | 1.0 | 7.5 |
| 0 | 0.5 | 0 | 0 | 1.0 | 0 | 1.5 |

**References**

1. Children's Otolaryngology Committee of the Pediatric Branch of the Chinese Medical Doctor Association, Pediatric Allergic Committee of Chinese Maternal and Child Health Association, Otolaryngology-Head and Neck Surgery Branch of Asia-Pacific Association of Medicine and Bio-Immunology. Expert consensus on clinical diagnosis and management of adenoid hypertrophy in children [Chinese]. Chin J Pract Pediatr. 2025;40(2):89-95+99. [doi: 10.19538/j.ek2025020601]
2. Ahmad Z, Krüger K, Lautermann J, Lippert B, Tenenbaum T, Tigges M, et al. Adenoid hypertrophy—diagnosis and treatment: the new S2k guideline. HNO. 2023 Aug;71(Suppl 1):67-72. [doi: 10.1007/s00106-023-01299-6]
