# Supplementary material for: Videos on Bilibili, TikTok, and Xiaohongshu as Sources of Medical Information on Adenoid Hypertrophy: Cross-Sectional Content Analysis
Source: JMIR Form Res. 2026 Jun 18;10:e82923. doi: 10.2196/82923 (PMC13278250; doi:10.2196/82923)
Supplement: Multimedia Appendix 2 [file formative-v10-e82923-s002.docx]

**The Patient Education Materials Assessment Tool (PEMAT)**

**Understandability.**

|  | Item | Response Options | Rating |
| --- | --- | --- | --- |
| Topic: Content | | | |
| 1 | The material makes its purpose completely evident. | Disagree=0, Agree=1 |  |
| Topic: Word Choice & Style | | | |
| 3 | The material uses common, everyday language. | Disagree=0, Agree=1 |  |
| 4 | Medical terms are used only to familiarize audience with the terms. When used, medical terms are defined. | Disagree=0, Agree=1 |  |
| 5 | The material uses the active voice. | Disagree=0, Agree=1 |  |
| Topic: Organization | | | |
| 8 | The material breaks or "chunks" information into short sections. | Disagree=0, Agree=1,  Very short material=N/A |  |
| 9 | The material’s sections have informative headers. | Disagree=0, Agree=1,  Very short material=N/A |  |
| 10 | The material presents information in a logical sequence. | Disagree=0, Agree=1 |  |
| 11 | The material provides a summary. | Disagree=0, Agree=1,  Very short material=N/A |  |
| Topic: Layout & Design | | | |
| 12 | The material uses visual cues (e.g., arrows, boxes, bullets, bold, larger font, highlighting) to draw attention to key points. | Disagree=0, Agree=1, Video=N/A |  |
| 13 | Text on the screen is easy to read. | Disagree=0, Agree=1,  No text or all text is narrated=N/A |  |
| 14 | The material allows the user to hear the words clearly (e.g., not too fast, not garbled). | Disagree=0, Agree=1,  No narration=N/A |  |
| Topic: Use of Visual Aids | | | |
| 18 | The material uses illustrations and photographs that are clear and uncluttered. | Disagree=0, Agree=1,  No visual aids=N/A |  |
| 19 | The material uses simple tables with short and clear row and column headings. | Disagree=0, Agree=1,  No tables=N/A |  |

Total Points: _____________

Total Possible Points: _____________

Understandability Score (%): _____________

(Total Points / Total Possible Points x 100)

**Actionability**

|  | Item | Response Options | Rating |
| --- | --- | --- | --- |
| 20 | The material clearly identifies at least one action the user can take. | Disagree=0, Agree=1 |  |
| 21 | The material addresses the user directly when describing actions. | Disagree=0, Agree=1 |  |
| 22 | The material breaks down any action into manageable, explicit steps. | Disagree=0, Agree=1 |  |
| 25 | The material explains how to use the charts, graphs, tables, or diagrams to take actions. | Disagree=0, Agree=1,  No charts, graphs, tables, diagrams=N/A |  |

Total Points: _____________

Total Possible Points: _____________

Actionability Score (%): _____________

(Total Points / Total Possible Points x 100)
