# Supplementary material for: Videos on Bilibili, TikTok, and Xiaohongshu as Sources of Medical Information on Adenoid Hypertrophy: Cross-Sectional Content Analysis
Source: JMIR Form Res. 2026 Jun 18;10:e82923. doi: 10.2196/82923 (PMC13278250; doi:10.2196/82923)
Supplement: Multimedia Appendix 3 [file formative-v10-e82923-s003.docx]

**Description of the modified DISCERN score.**

| Criteria  (1 point for each) | Description |
| --- | --- |
| 1 | Is the video clear, concise, and understandable? |
| 2 | Are reliable sources of information used? (i.e., publication cited, speaker is specialist) |
| 3 | Is the information presented balanced and unbiased? |
| 4 | Are additional sources of information listed for patient reference? |
| 5 | Are areas of uncertainty/controversy mentioned? |
