# Supplementary material for: Videos on Bilibili, TikTok, and Xiaohongshu as Sources of Medical Information on Adenoid Hypertrophy: Cross-Sectional Content Analysis
Source: JMIR Form Res. 2026 Jun 18;10:e82923. doi: 10.2196/82923 (PMC13278250; doi:10.2196/82923)
Supplement: Multimedia Appendix 4 [file formative-v10-e82923-s004.docx]

**Description of the Global Quality Score (GQS) scale**

| Scale | Description |
| --- | --- |
| Poor quality (1 point) | Poor quality and poor flow of the site, most information missing, not at all useful for patients |
| Generally poor quality (2 point) | Generally poor quality and poor flow, some information listed but many important topics missing, of very limited use to patients |
| Moderate quality (3 point) | Moderate quality, sub-optimal flow, some important information is adequately discussed but others poorly discussed, somewhat useful for patients |
| Good quality (4 point) | Good quality and generally good flow, most of the relevant information is listed, but some topics not covered, useful for patients |
| Excellent quality (5 point) | Excellent quality and excellent flow, very useful for patients |
