# Supplementary material for: Videos on Bilibili, TikTok, and Xiaohongshu as Sources of Medical Information on Adenoid Hypertrophy: Cross-Sectional Content Analysis
Source: JMIR Form Res. 2026 Jun 18;10:e82923. doi: 10.2196/82923 (PMC13278250; doi:10.2196/82923)
Supplement: Multimedia Appendix 5 [file formative-v10-e82923-s005.docx]

**Representative examples of video content themes and potentially misleading or non-evidence-based claims identified during content analysis**

| Video ID | Platform | Uploader identity | Content theme | Misinformation type | Representative statement (verbatim or paraphrased) |
| --- | --- | --- | --- | --- | --- |
| 11 | Bilibili | Parent of a child patient | Treatment decision | Anti-surgery | “Surgery is not recommended for adenoid hypertrophy; you will regret it if you remove the adenoids.” |
| 71 | Bilibili | Pediatrician | Complications / prognosis | Exaggeration of consequences | “Surgery should be done early; otherwise, the child will become ugly, and growth, intelligence, and learning will be affected.” |
| 81 | Bilibili | Pediatrician | Complications / prognosis | Exaggeration of consequences | “Early surgery is necessary; otherwise, adenoid facies will develop, and growth, intelligence, and learning will be impaired.” |
| 5 | TikTok (Douyin) | Pediatrician | Treatment | Promotion of non–evidence-based therapy | “Surgery is not needed for adenoid hypertrophy; several Chinese herbal formulas can cure it.” |
| 13 | TikTok (Douyin) | Otorhinolaryngologist | Treatment decision | Overgeneralized recommendation“Children with adenoid | “Children with adenoid hypertrophy should have surgery early.” |
| 57 | TikTok (Douyin) | Otorhinolaryngologist | Treatment | Promotion of non–evidence-based therapy | “90% obstruction without surgery? You can try Chinese herbal medicine instead.” |
| 20 | Xiaohongshu | Parent of a child patient | Treatment | Lifestyle-based unsupported claims | “Adenoidectomy is not recommended. The real ways to reduce adenoid swelling are just two: more exercise and eating less spicy food.” |
| 25 | Xiaohongshu | Parent of a child patient | Treatment | Folk remedy | “Don’t you believe that adenoid hypertrophy can be treated without surgery? Tuina massage can help children avoid surgery.” |
| 65 | Xiaohongshu | Science blogger | Treatment | Promotion of non–evidence-based therapy | “Traditional Chinese Medicine is more reliable for adenoid hypertrophy; dietary therapy is recommended.” |
