# Supplementary material for: Videos on Bilibili, TikTok, and Xiaohongshu as Sources of Medical Information on Adenoid Hypertrophy: Cross-Sectional Content Analysis
Source: JMIR Form Res. 2026 Jun 18;10:e82923. doi: 10.2196/82923 (PMC13278250; doi:10.2196/82923)
Supplement: Multimedia Appendix 6 [file formative-v10-e82923-s006.docx]

****Table S5.** Stepwise linear regression intermediate models (Models 1–3) for GQS prediction (N = 220)**

| Model | Predictor | B | SE | *β* | *t* | *P* | 95% CI | VIF |
| --- | --- | --- | --- | --- | --- | --- | --- | --- |
| 1 | (Constant) | 0.426 | 0.183 |  | 2.326 | .021 | 0.065 – 0.786 |  |
|  | PEMAT‑U | 0.027 | 0.002 | 0.610 | 11.360 | < .001 | 0.022 – 0.032 | 1.000 |
| 2 | (Constant) | 0.257 | 0.182 |  | 1.415 | .158 | –0.101 – 0.616 |  |
|  | PEMAT‑U | 0.024 | 0.002 | 0.545 | 10.018 | < .001 | 0.019 – 0.029 | 1.097 |
|  | PEMAT‑A | 0.006 | 0.001 | 0.218 | 4.010 | < .001 | 0.003 – 0.009 | 1.097 |
| 3 | (Constant) | 0.136 | 0.179 |  | 0.759 | .449 | –0.218 – 0.490 |  |
|  | PEMAT‑U | 0.024 | 0.002 | 0.529 | 9.986 | < .001 | 0.019 – 0.028 | 1.104 |
|  | PEMAT‑A | 0.007 | 0.001 | 0.245 | 4.603 | < .001 | 0.004 – 0.009 | 1.118 |
|  | Video duration (s) | 0.001 | 0.000 | 0.193 | 3.791 | < .001 | 0.000 – 0.001 | 1.020 |

Note: Model 4 (final model) is presented in the main text (Table 5). Abbreviations: SE, standard error; CI, confidence interval; VIF, variance inflation factor.
